# Considering Angle Selection When Using Ultrasound Electrode Displacement Elastography to Evaluate Radiofrequency Ablation of Tissues

**DOI:** 10.1155/2014/764320

**Published:** 2014-05-27

**Authors:** Jingjing Xia, Qiang Li, Pin-Yu Chen, Zhuhuang Zhou, Chiao-Yin Wang, Hao-Li Liu, Jianfu Teng, Po-Hsiang Tsui

**Affiliations:** ^1^School of Electronic Information Engineering, Tianjin University, Tianjin 300072, China; ^2^Department of Medical Imaging and Radiological Sciences, College of Medicine, Chang Gung University, 259 Wen-Hwa 1st Road, Kwei-Shan, Taoyuan, Taoyuan County 33302, Taiwan; ^3^Biomedical Engineering Center, College of Life Science and Bioengineering, Beijing University of Technology, Beijing 100124, China; ^4^Graduate Institute of Clinical Medical Sciences, College of Medicine, Chang Gung University, Taoyuan 33302, Taiwan; ^5^Department of Electrical Engineering, Chang Gung University, Taoyuan 33302, Taiwan; ^6^Institute for Radiological Research, Chang Gung Memorial Hospital at Linkou, Chang Gung University, Taoyuan 33302, Taiwan

## Abstract

Percutaneous radiofrequency ablation (RFA) is a minimally invasive treatment to thermally destroy tumors. Ultrasound-based electrode-displacement elastography is an emerging technique for evaluating the region of RFA-induced lesions. The angle between the imaging probe and the RFA electrode can influence electrode-displacement elastography when visualizing the ablation zone. We explored the angle effect on electrode-displacement elastography to measure the ablation zone. Phantoms embedded with meatballs were fabricated and then ablated using an RFA system to simulate RFA-induced lesions. For each phantom, a commercial ultrasound scanner with a 7.5 MHz linear probe was used to acquire raw image data at different angles, ranging from 30° to 90° at increments of 10°, to construct electrode-displacement images and facilitate comparisons with tissue section images. The results revealed that the ablation regions detected using electrode-displacement elastography were highly correlated with those from tissue section images when the angle was between 30° and 60°. However, the boundaries of lesions were difficult to distinguish, when the angle was larger than 60°. The experimental findings suggest that angle selection should be considered to achieve reliable electrode-displacement elastography to describe ablation zones.

## 1. Introduction

Liver cancer is a major public health problem worldwide, and, based on data compiled by the National Center for Health Statistics in 2013 [1], the mortality rate of liver cancer has steadily increased over the previous 3 decades. Several alternative therapeutic methods are available for hepatic tumors in addition to chemotherapy, such as surgical resection or liver transplants [2–4]. Because patients with poor liver function or distributed tumors are unsuited to undergoing surgical resection and liver transplant, radiofrequency ablation (RFA) is used as a minimal invasive strategy for liver tumor treatment. RFA is one of the most popular choices for hepatic tumor treatments [5, 6].

During RFA, a radiofrequency (RF) needle electrode is inserted into the tumor to deliver a strong alternating electrical current. This current agitates ions and produces a temperature increase that induces the coagulation necrosis of tissues surrounding the electrode. Paramount to the success of RFA therapy is that the ablation region adequately covers the entire tumor to ensure a complete destruction of cancer cells. Thus, developing imaging modalities that accurately portray the ablation zones is crucial [7]. Ultrasound is the most frequently used modality to monitor RFA because of its portability, low cost, real-time capability, and compatibility with other medical equipment. RFA-induced necrosis tissue has a greater stiffness compared with the background untreated tissues [8–11]. Consequently, ultrasound elastography, a functional ultrasound image that reflects tissue stiffness, might provide reliable, high-contrast images for monitoring the ablation region [12, 13]. Ultrasound elastography can be classified into 3 types: (1) those that apply a quasistatic compression and estimate the resulting components of the strain tensor [12–18]; (2) those that apply low-frequency vibration with ultrasound Doppler detection of velocities of perturbed reflectors [19–21]; and (3) those that use acoustic-radiation force [8, 9, 22].

In the current clinical diagnosis, the type I elastography previously mentioned (i.e., strain imaging) is widely used because of its popularity and compatibility with the conventional pulse-echo ultrasound system. However, when applying the ultrasound strain image to describe the ablation zone, using the transducer to compress tissues (external compression) might be difficult in producing internal strain in liver tissue in vivo. To address this concern, Varghese et al. [8, 9, 23, 24] proposed adopting perturbations of the RF electrode as the mechanical stimulus to induce tissue displacements for elastography. The incremental displacements applied to the electrode provide localized compression in the thermal lesion [25]. Nightingale et al. [26] also employed localized compression in using acoustic-radiation force to introduce perturbations in the targeted tissue region. Bharat et al. [23, 24, 27–30] performed both phantom and in vivo animal experiments to validate that electrode-displacement elastography is capable of providing high-contrast images, using widely available commercial ultrasound systems that can be used to assess the extent of thermal ablation zones.

The aforementioned studies placed the imaging probe parallel to the RF electrode when performing electrode-displacement strain imaging for monitoring RFA to measure the maximum strain of the tissue for imaging (the angle between the imaging transducer and the RF electrode should be as small as possible). However, such an arrangement might limit the clinical applications of electrode-displacement strain imaging. Tumors can form at any depth of liver tissue and thus might be outside the effective range of electrode-displacement strain imaging based on the suggested minimum angle. Monitoring the RFA of tumors at various depths and locations might necessitate using various angle arrangements for electrode-displacement strain imaging in future clinical applications. Establishing a multiangle-based electrode-displacement strain imaging is a long-term goal. Therefore, the effects of angles on electrode-displacement strain imaging must be clarified. Because of the absence of research on the effects of angles on electrode-displacement elastography, we investigated the effects of angles on using electrode-displacement strain imaging to monitor RFA.

## 2. Materials and Methods

### 2.1. Experimental Setup

**Figure 1:**
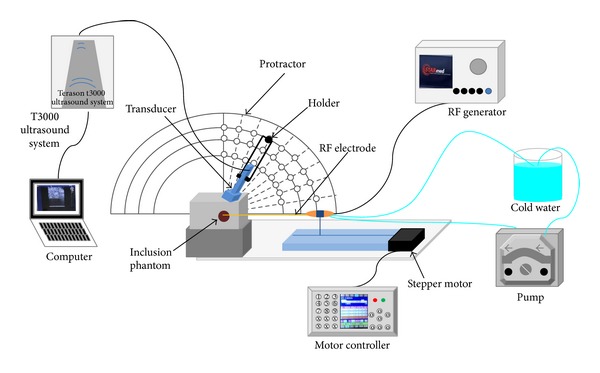
Schematic illustration of the experimental setup for exploring the angle effect on using electrode-displacement elastography to monitor RFA.

The experimental setup (Figure 1) consisted of 4 major parts: a clinical RFA system, a commercial ultrasound imaging system, a protractor, and a stepper motor system. The RFA system (Model VIVA RF Generator, STARmed Co. Ltd., Goyang, Gyeonggi, Republic of Korea) included a cool-tip RF electrode (Model 17-20V15-40, STARmed Co.) and an RF generator for providing an adjustable RF output power ranging from 0 to 200 W, with an operation frequency of 480 kHz. The RFA system also included a peristaltic pump, which was used to deliver a constant flow of cold saline solution with a mixture of ice to the electrode tip for preventing the electrode from overheating, resulting in tissue carbonization. The ultrasound imaging system (Model 3000, Terason, Burlington, MA, USA) was equipped with a 7.5 MHz linear array transducer (Model 12L5V, Terason), which was used to monitor RFA and acquire raw backscattered RF data at a sampling rate of 37.5 MHz for subsequent electrode-displacement imaging. The protractor was used to calibrate the angle between the imaging transducer and the RF electrode from 30° to 90° in increments of 10°. The stepper motor system (Model ELS2XE003-KP, Oriental Motor, Tokyo, Japan) with a self-made electrode holder was used to move the RF electrode back and forth for inducing displacement of tissue samples.

### 2.2. Material Preparation and Experimental Procedure

In order to explore the angle effect on electrode-displacement elastography, we referred to a previous study [23] to construct agar phantoms embedded with meatball inclusions (*n* = 14). In practice, an intermediate transition zone will appear between the unablated and the RFA-induced necrotic tissues, making it difficult to accurately identify the ablation region. For this reason, we used phantoms with meatballs instead of whole tissue samples in the experiments to provide a standard ablation size for comparison (necrosis following RFA only occurs in part of the inclusion). The diameter of the embedded inclusions ranged from 11.09 to 19.12 mm, with a mean and standard deviation of 140.19 ± 51.48 mm^2^. After embedding the meatball in the agar phantom, RF electrode was inserted into the meatball inclusion to ablate the tissue. We have confirmed that the settings (ablation time and power, described in the next paragraph) used for RFA allow the ablation of the meatball inclusion completely. In the phantom, we added graphite powder with diameter <20 *μ*m (Model 282863, Sigma-Aldrich, St. Louis, MO, USA) to simulate scatterers and image speckle in the background. The previous study [23] revealed that the agar phantom embedded with the ablated inclusion can produce the difference of the stiffness between the phantom background and the ablation zone for testing the electrode-displacement elastography.

For each inclusion phantom, RFA was performed at the default automatic mode, which started at 50 and automatically increased by 10 W/min until the first RF pulse paused because of high tissue impedance before generating a sequence of RF pulses as a time function. The treatment time for the automatic mode was 12 min. Following the ablation, the inclusion phantom cooled for 12 min. The stepper motor system subsequently controlled the RF electrode to produce tissue displacements of 1 mm. An image frame was acquired when the RF electrode moved forward 0.1 mm. In the range of 1 mm, a total of 10 image frames were acquired from the ultrasound system for electrode-displacement imaging. These measurements were repeated for angles from 30° to 90°.

### 2.3. Algorithm for Electrode-Displacement Elastography

**Figure 2:**
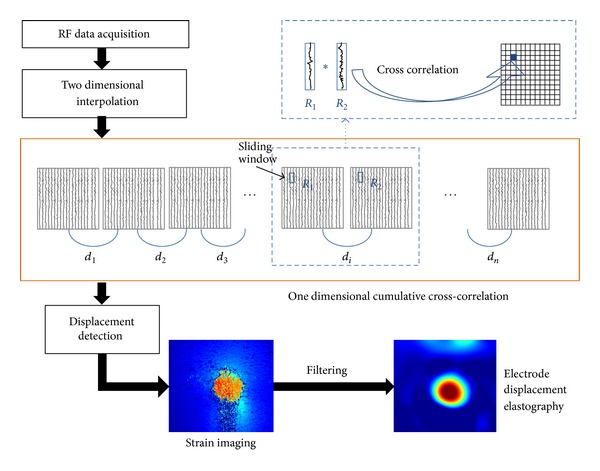
Flow chart of the algorithm for constructing the electrode-displacement elastography.

Figure 2 shows the flow chart of the algorithm for electrode-displacement imaging. One-dimensional cross-correlation analysis of 2 consecutively acquired images of RF data following interpolation was performed using a 3 mm long window with an overlap ratio of 75% to obtain the tissue-displacement image. The strain images were obtained from the axial differentiation of the displacement image. To remove noise and smooth images, we applied a 3 × 3 median filter and an 8-order 2-dimensional Butterworth low-pass filter at the cutoff frequency of 5 MHz to process the strain image.

### 2.4. Data Comparison

For each inclusion phantom, the size of the meatball was measured as the ground truth of the ablation zone. We used the ImageJ free image analysis software to outline the ablation zone detected in the electrode-displacement image. At first, a square region of interest only containing the ablation zone detected in the image was used as a subimage. Then, we used the ImageJ software to adjust the threshold color of the subimage, until the contour of the ablation zone was well detected. By calibration using the scale of the electrode-displacement image, ImageJ software automatically measured the size of the segmented ablation area. The ablation sizes obtained from the sample measurements and the electrode-displacement image were compared using linear regression in the form of *y* = *y*_0_ + *ax* to calculate the correlation coefficient, thereby determining the angle effect on using electrode-displacement imaging to visualize the ablation zones.

## 3. Results

**Figure 3:**
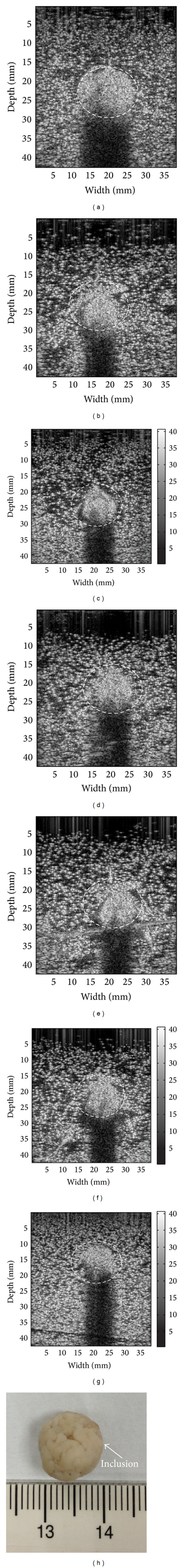
B-mode images obtained at different angles: (a) angle = 30°, (b) angle = 40°, (c) angle = 50°, (d) angle = 60°, (e) angle = 70°, (f) angle = 80°, and (g) angle = 90°; (h) embedded meatball in the inclusion phantom.

**Figure 4:**
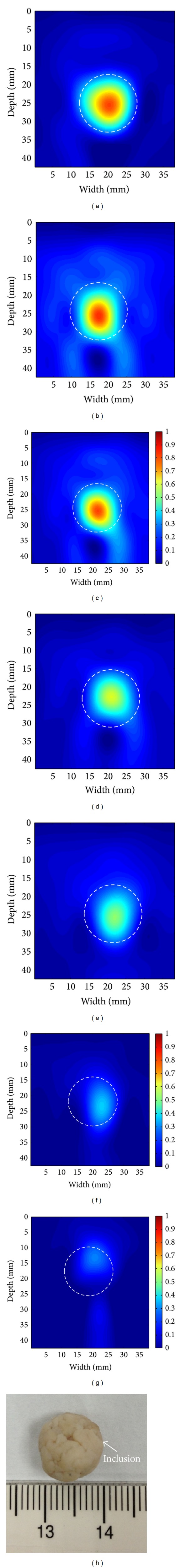
Electrode-displacement images obtained at different angles: (a) angle = 30°, (b) angle = 40°, (c) angle = 50°, (d) angle = 60°, (e) angle = 70°, (f) angle = 80°, and (g) angle = 90°; (h) embedded meatball in the inclusion phantom.

**Figure 5:**
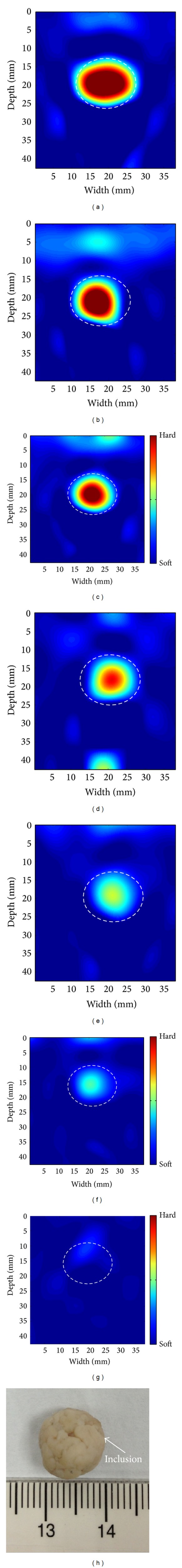
Electrode-displacement strain images obtained at different angles: (a) angle = 30°, (b) angle = 40°, (c) angle = 50°, (d) angle = 60°, (e) angle = 70°, (f) angle = 80°, and (g) angle = 90°; (h) embedded meatball in the inclusion phantom.

Figure 3 shows B-mode images of the inclusion phantom obtained at various angles ranging from 30° to 90° after RFA. The conventional B-mode image indicated no ability to identify the change in tissue stiffness after RFA. Figures 4 and 5 present the corresponding electrode-displacement and strain images. The ablation zone (the inclusion region) was clearly described using electrode-displacement strain imaging, when the angle between the transducer and the electrode was smaller than 60°. However, when the angle exceeded 60°, the ablation zone detected in the electrode-displacement elastography gradually disappeared. This result indicated that using electrode-displacement elastography to monitor RFA is highly dependent on the angle between the transducer and the RF electrode.

**Figure 6:**
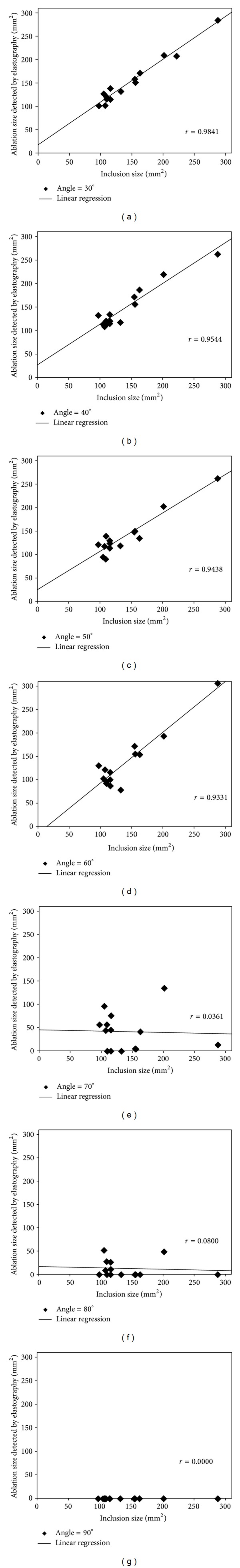
Comparisons between the meatball sizes and the ablation zones detected by electrode-displacement strain imaging (*n* = 14). (a) angle = 30°, (b) angle = 40°, (c) angle = 50°, (d) angle = 60°, (e) angle = 70°, (f) angle = 80°, and (g) angle = 90°. The correlation coefficient decreases with increasing the angle between the transducer and the RF electrode. No significant correlation was found when the angle was larger than 60°.

To further confirm the experimental findings, we explored the relationship between the inclusion sizes and the ablation zones detected using the electrode-displacement strain image (Figure 6).  Table 1 summarizes the comparison results. When the angles were smaller than 60°, the correlation coefficients obtained from the linear regression were larger than 0.9 and the error reduced to less than 15%. However, the correlation coefficient reduced to less than 0.05 and the error increased when the angle was larger than 60°.

## 4. Discussion

### 4.1. The Significance of This Study

Electrode-displacement strain imaging involves using the RF electrode as a displacement device, which provides localized compression in the region of interest. This displacement mechanism offers the possibility of in vivo implementation because it is difficult to produce tissue strain through conventional ultrasound elastography using external compression techniques. Before using the electrode-displacement strain image as a reliable clinical tool for applications, certain issues require further investigation. One critical issue is the effect of the imaging angle between the ultrasound transducer and the RF electrode on the performance of electrode-displacement strain imaging in monitoring RFA. We clarified the angle issue and suggested a prerequisite angle <60° for a successful formation of electrode-displacement strain imaging.

### 4.2. Physical Meaning of Electrode-Displacement Imaging

During the RFA procedure, the temperature in the tissue typically reaches boiling temperature. The ablation zone behaves similarly to a hyperechoic region in the ultrasound image because of bubble formation under such high-temperature ablation. Previous studies have observed that bubbles insonified through ultrasound with sufficient intensity and duration can emit various subharmonic components [31] and other low frequency emissions [32, 33]. Based on this viewpoint, Winkler and Adam [34] proposed using acoustic emissions at low frequencies from the RFA-induced bubbles for assessing RFA monitoring.

However, by decreasing the tissue temperature following the RFA procedure, the dissolved bubbles cause the hyperechoic region to disappear, and the ablation zone typically manifests as a mixed echogenicity zone in the B-mode image. Tissue stiffness subsequently becomes crucial for evaluating the performance of RFA treatment. RFA-induced necrosis zones are considerably stiffer than untreated regions. In principle, the displacement detected in ablation zones should be small compared with that of untreated tissues. Although previous studies have observed that the ablation zone produces small tissue displacement [8, 9], our experiment results revealed the opposite phenomenon. The results of electrode-displacement imaging (Figure 4) showed that the ablation zone exhibited large displacement. We assumed that the tissue displacement might rely on the adhesion force of the ablation tissue to the RF electrode. In our experiments, RFA was operated at the default mode of 50 W to produce an extremely high temperature. We observed that the RF electrode strongly adhered to the necrotic tissue after RFA was completed. In this condition, the necrotic inclusion in the phantom produced a substantial axial displacement, when the RF electrode moved back and forth. The degree of adhesion of the RF electrode to the ablated tissue might further rely on the ablation temperature (or power), water content in tissues, the degree of necrosis, and the tissue type. In other words, how to identify “soft” and “hard” in electrode-displacement elastography in various clinical applications can be redefined.

### 4.3. Angle Effect on Electrode-Displacement Strain Imaging

In the following discussion, we attempt to clarify why a large angle between the ultrasound transducer and the RF electrode is unfavorable to forming electrode-displacement strain imaging. This can be explained using a sample discussion from mechanics. In conventional electrode-displacement strain imaging, the RF electrode is controlled by a one-dimensional step motor to produce axial displacements, resulting in axial strain of tissues. When the angle between the ultrasound transducer and the RF electrode is small, the echo time-shift detected in the plane of ultrasound image can sensitively reflect tissue axial strain, producing a reliable electrode-displacement strain image. When the angle is large, the ultrasound image detects the lateral vector of the axial strain in the tissue. The echo time-shift corresponding to the lateral vector of the axial strain detected in the ultrasound image decreases with an increasing angle, rendering it difficulty to form the electrode-displacement strain image.

### 4.4. Strengths and Limitations of Electrode-Displacement Strain Imaging

Using ultrasound elastography to describe the necrosis region generated from RFA will be a trend in future clinical diagnosis. Both acoustic-radiation force impulse (ARFI) imaging and supersonic shear-wave imaging (SSI) are novel techniques for ultrasound elastography. A previous study used ARFI elastography and SSI for guiding and assessing thermal ablation [35, 36]. ARFI elastography was developed to measure shear-wave velocity, which is related directly to tissue stiffness. The ARFI system measures shear-wave velocity by repeating push pulses and detecting pulses across a region of interest. Compared with ARFI, a shear-wave elastography system visualizes absolute tissue stiffness. SSI entails generating a remote radiation force by using focused ultrasonic beams (i.e., pushing beams). Each pushing beam generates a remote vibration that propagates a transient shear wave. Several pushing beams at increasing depths are transmitted to generate a quasiplane shear-wave front that propagates throughout the entire imaging area. After generating the shear wave, an ultrafast echographic imaging sequence is performed to acquire successive raw data at a high frame rate to estimate the shear-wave velocity and the shear modulus for imaging.

ARFI and SSI can provide superior boundary definition of structures relative to using conventional sonography alone. Using ARFI and SSI to monitor RFA does not require using the RF electrode to produce tissue strain (tissue strain is induced by pushing beams). Recent studies have also reported that using electrode perturbation can produce shear waves that are tracked at high frame rates for electrode vibration elastography [37–39]. However, ARFI and SSI have not yet been popularized in current commercial ultrasound systems. Therefore, electrode-displacement strain imaging remains strong and clinically crucial because it can be implemented using a conventional ultrasound B-scan system. Consequently, we suggested an appropriate angle for successfully forming electrode-displacement strain imaging as a crucial consideration in future clinical applications. The threshold angle also implies a possible limitation when using the electrode-displacement strain image for monitoring RFA in vivo. That is, electrode-displacement strain imaging is not necessary to have the ability for monitoring the RFA of tumors at various locations and depths.

### 4.5. Suggestions for Future Studies

We have two suggestions for future work. (i) In the current study, electrode-displacement strain imaging is based on one-dimensional cross-correlation analysis. Using a two-dimensional speckle tracking technique may improve the construction of electrode-displacement strain image using a large-angle arrangement. However, different algorithms may have different performances and efficiencies. Thus, the effects of various algorithms on electrode-displacement strain imaging need to be investigated and further compared. (ii) To mitigate the limitations of electrode-displacement strain imaging caused by the angle effect, we propose the possibility of combining electrode-displacement strain imaging and other imaging techniques by using the dual-transducer arrangement. One transducer can be responsible for electrode-displacement strain imaging at small angles to visualize the ablation zone, whereas the other transducer can be arranged at large angles to describe the ablation zone by implementing a certain imaging technique that is independent of the measurement angle (e.g., ultrasound Nakagami imaging proposed recently for monitoring RFA by modeling the envelope statistics [40]). In a clinical environment without advanced ultrasound systems, using a conventional grayscale ultrasound system combined with electrode-displacement strain imaging and other imaging techniques is a strategy for researchers to pursue.

## 5. Conclusion

This study explores the effects of angle on using electrode-displacement strain imaging to monitor RFA by performing experiments on inclusion phantoms. The primary contribution of this study is demonstrating that the angle required for successfully constructing electrode-displacement elastography should be smaller than 60°, allowing the ultrasound transducer to effectively reflect tissue axial strain caused by perturbations of the RF electrode. This experimental finding should be treated as a crucial consideration for future clinical applications and developments of electrode-displacement elastography.

## Figures and Tables

**Table 1:** Comparisons between the inclusion sizes and the ablation zones detected by electrode displacement strain imaging. The correlation coefficient decreases with increasing the angle between the transducer and the RF electrode. The correlation coefficient is smaller than 0.9 when the angle is larger than 60°. Data are expressed by mean ± standard deviation.

Angle	Meatball size (inclusion phantom)	Ablation size (electrode displacement strain image)	Error (%)	Correlation coefficient
30°	140.19 ± 51.48	152.87 ± 51.36	6.65 ± 1.51	0.9841
40°	140.19 ± 51.48	148.39 ± 46.75	10.13 ± 2.07	0.9544
50°	140.19 ± 51.48	139.73 ± 44.60	11.00 ± 1.86	0.9438
60°	140.19 ± 51.48	136.64 ± 59.77	13.02 ± 2.71	0.9331
70°	140.19 ± 51.48	41.12 ± 41.29	67.56 ± 6.88	0.0361
80°	140.19 ± 51.48	12.55 ± 18.80	89.92 ± 3.33	0.0800
90°	140.19 ± 51.48	N/A	100	0
